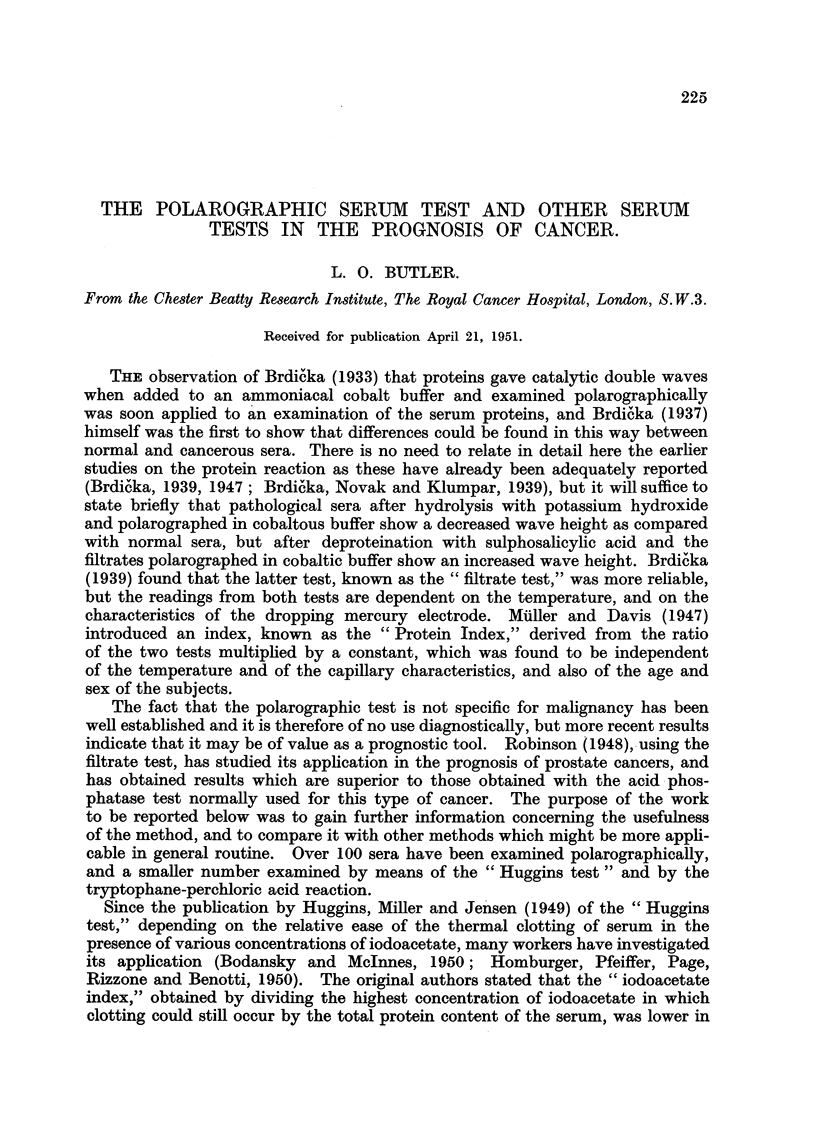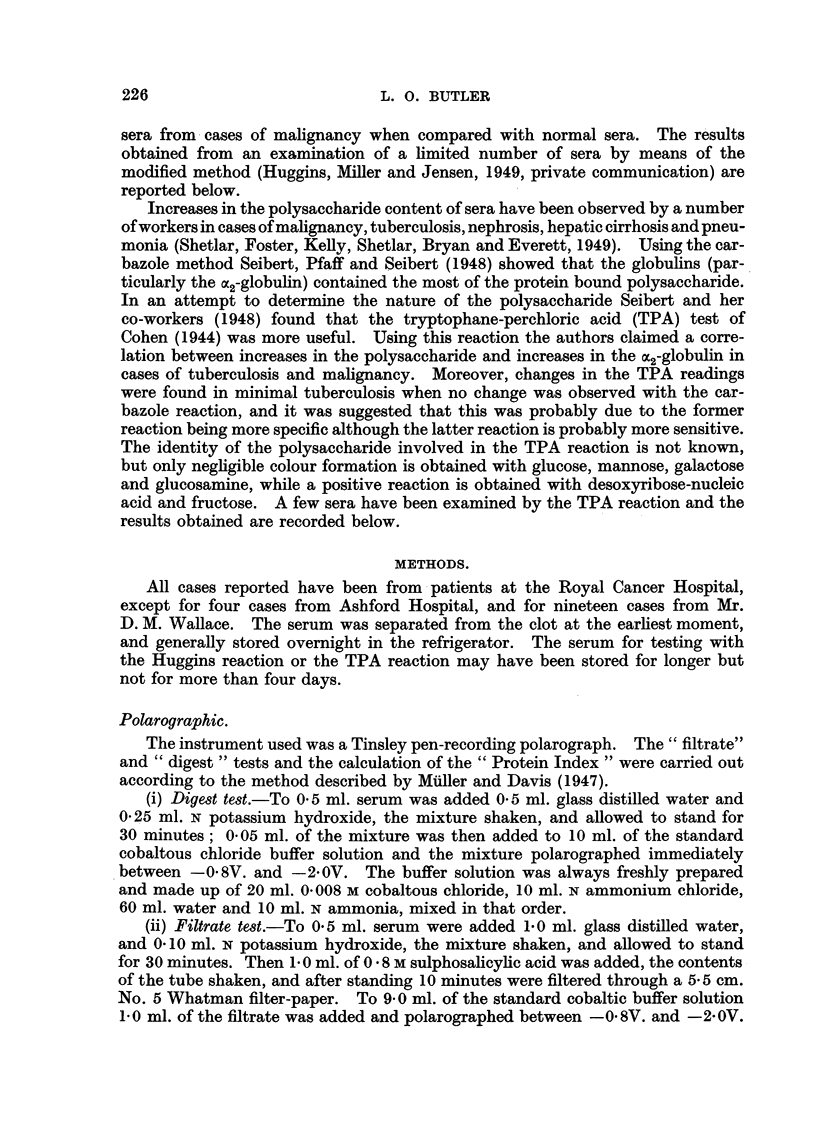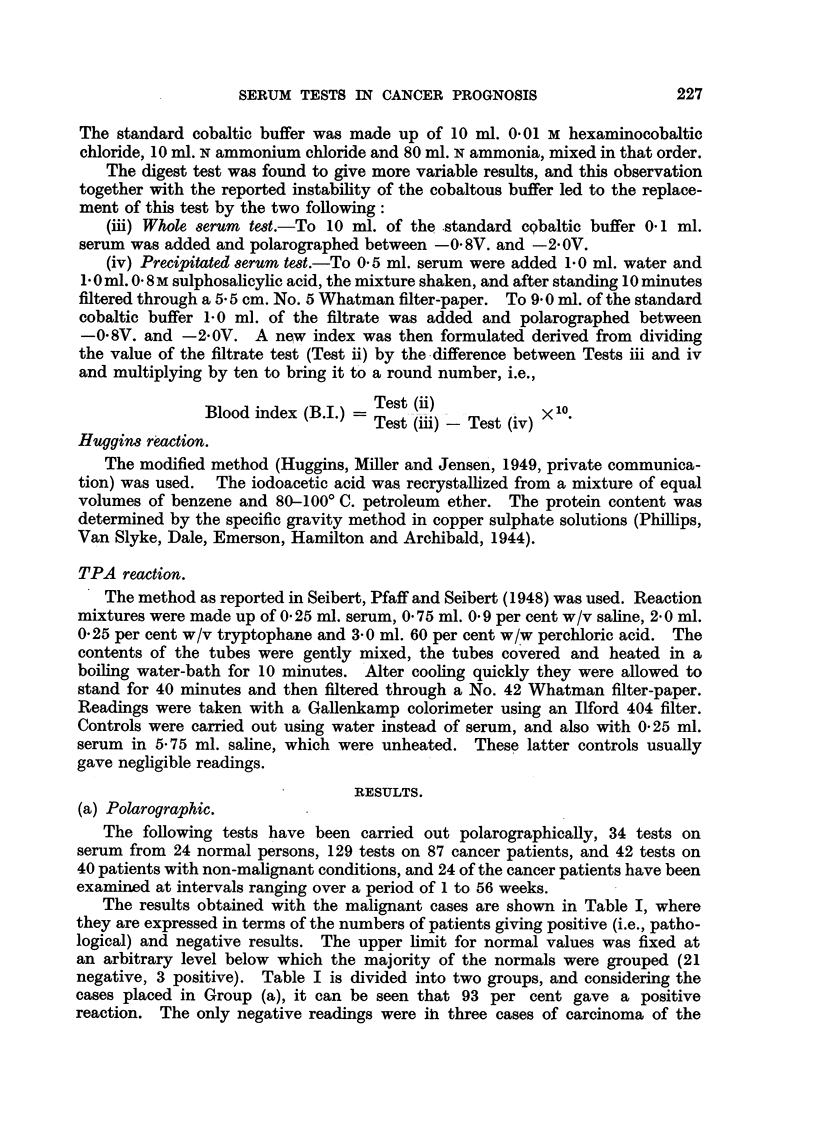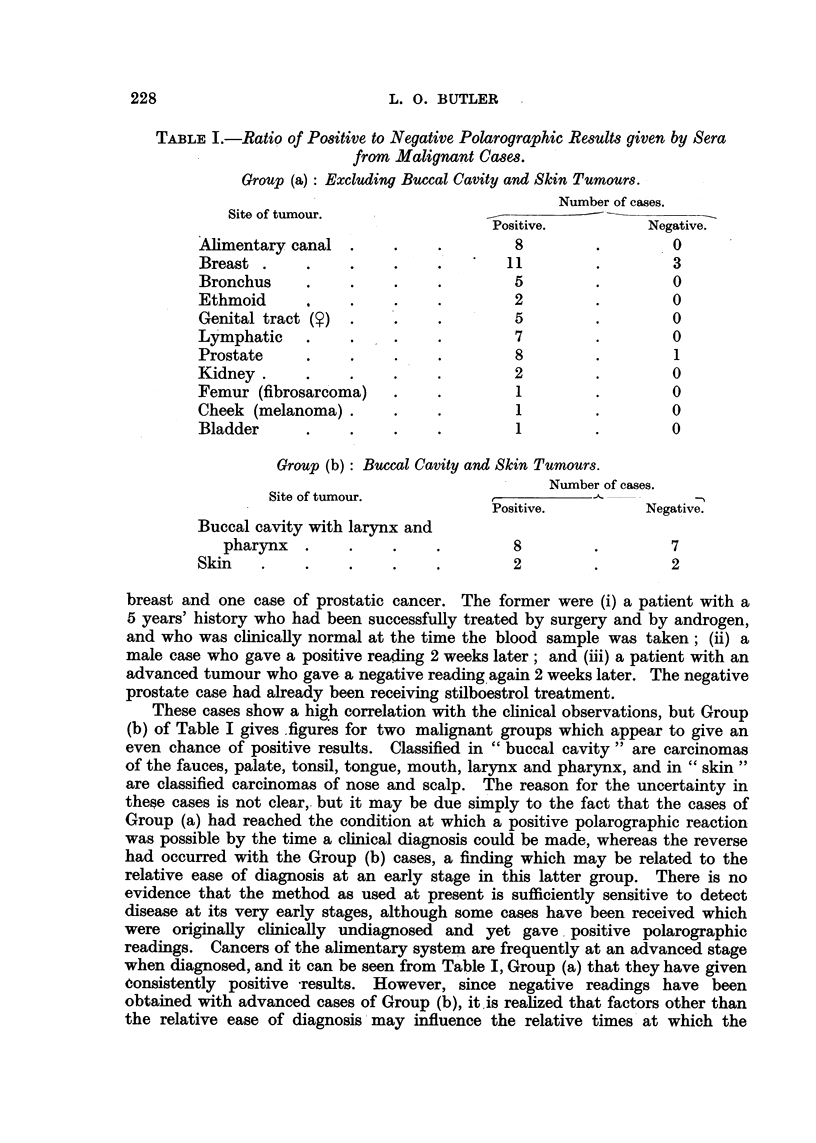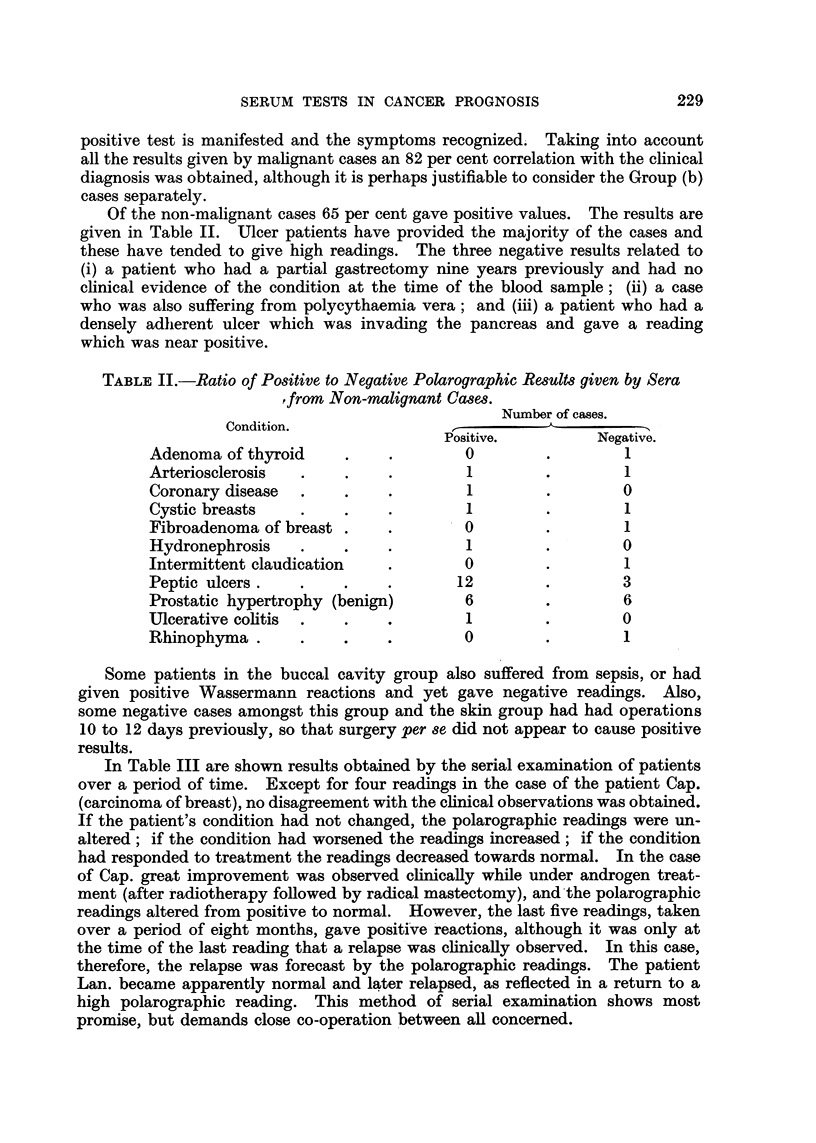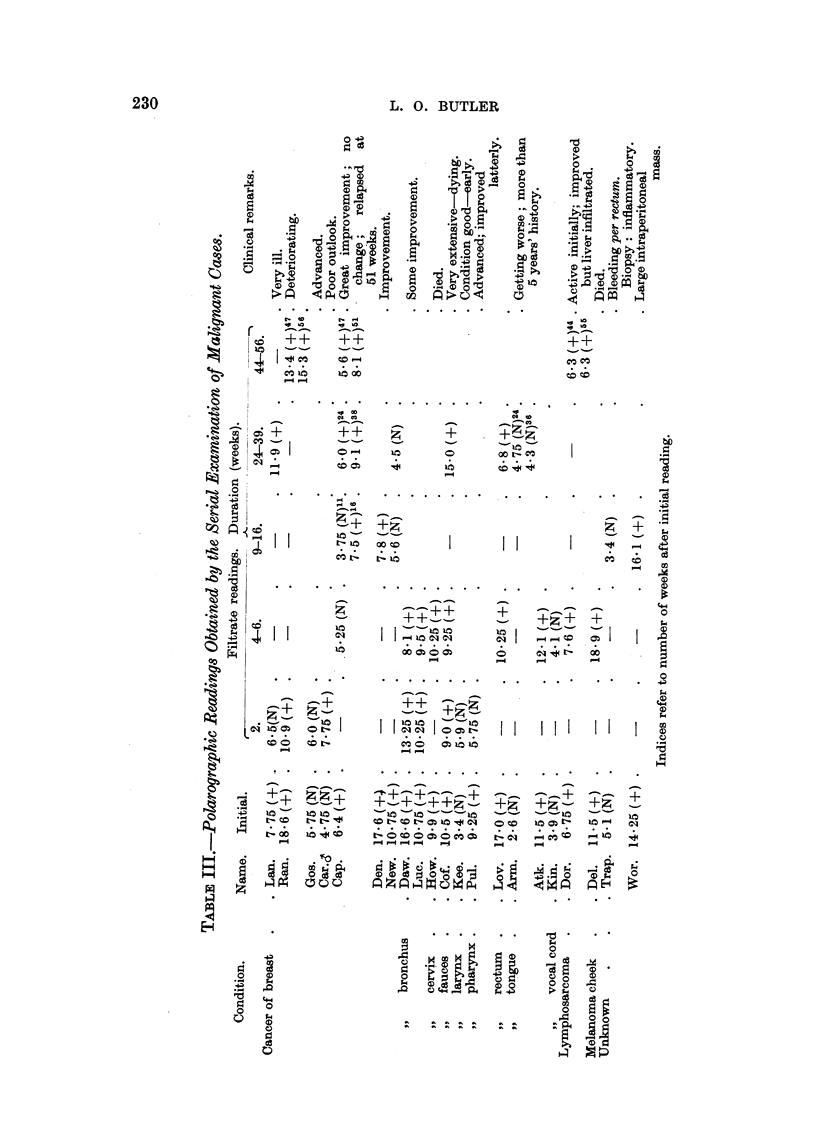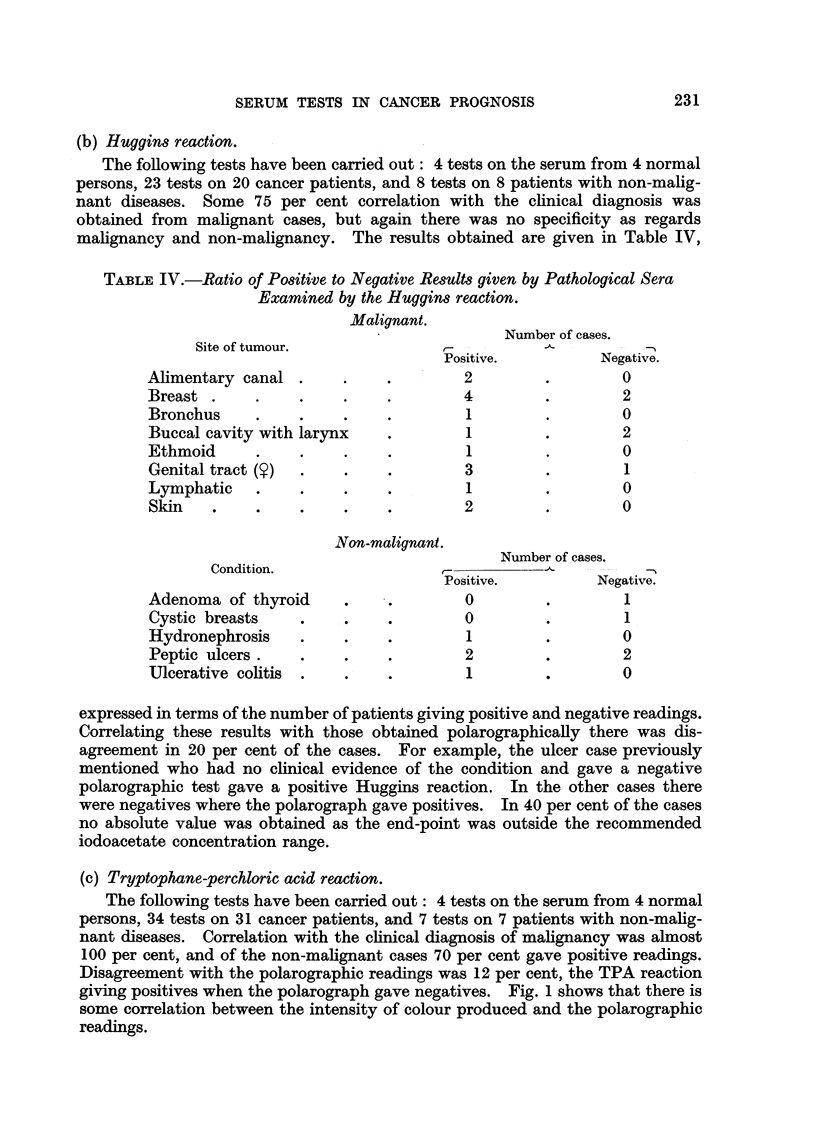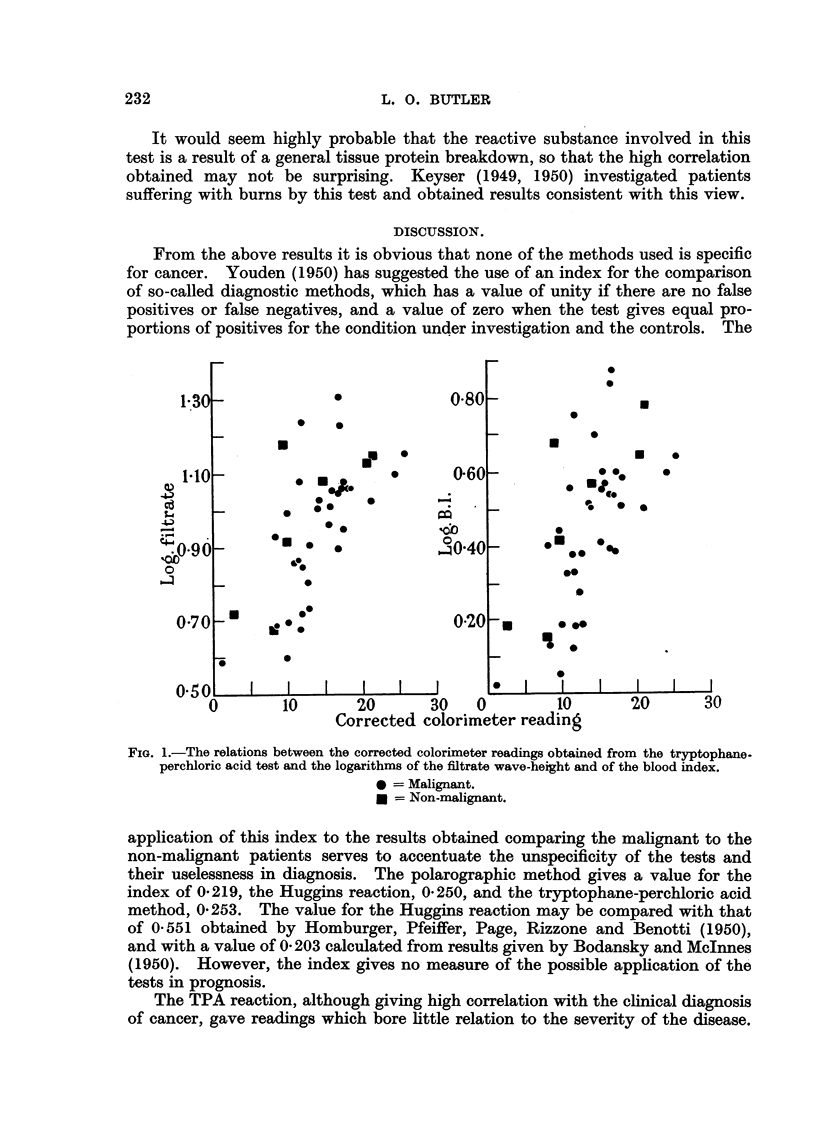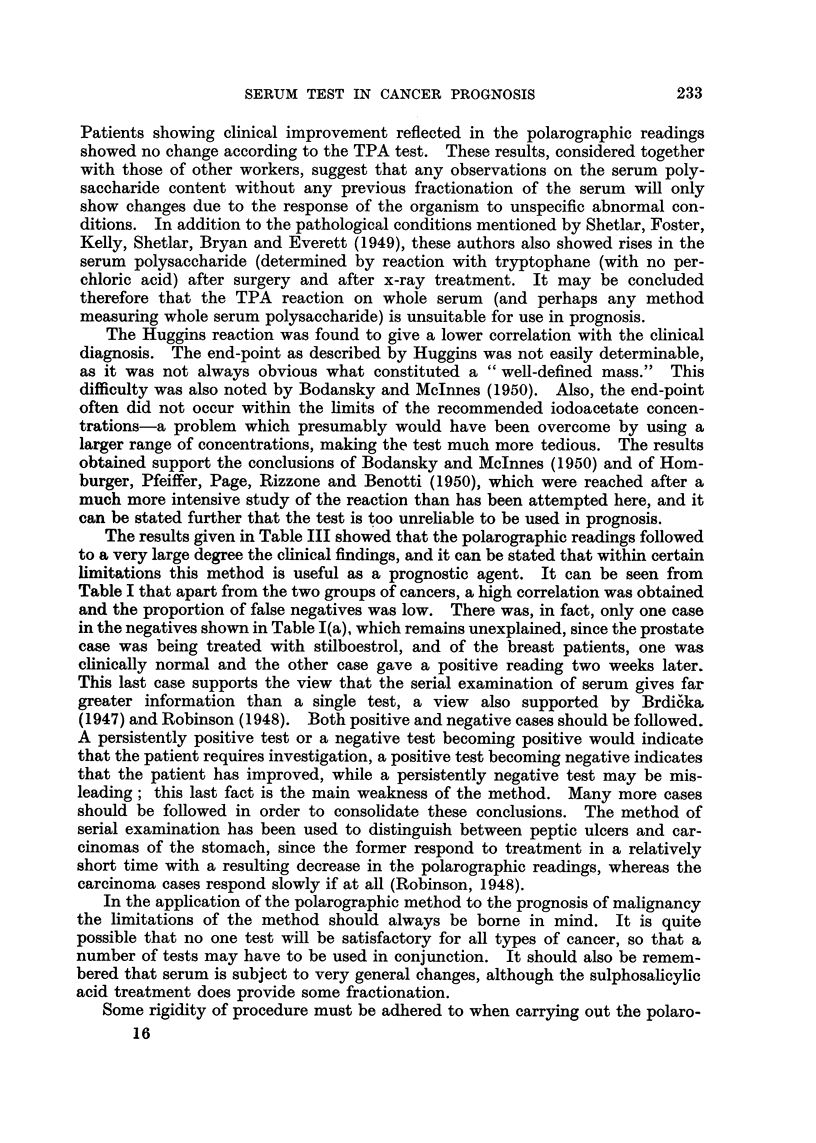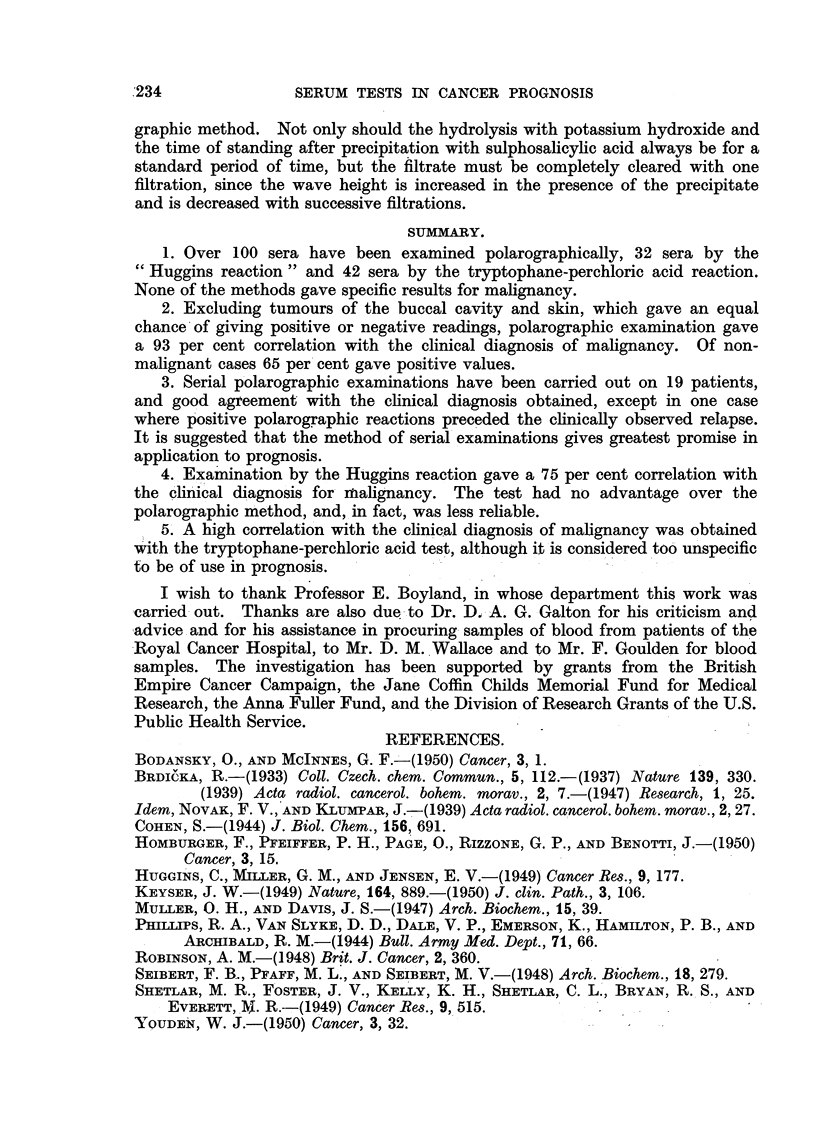# The Polarographic Serum Test and other Serum Tests in the Prognosis of Cancer

**DOI:** 10.1038/bjc.1951.23

**Published:** 1951-06

**Authors:** L. O. Butler


### THE POLAROGRAPHIC SERUM TEST AND OTHER SERUM TESTS IN THE PROGNOSIS OF CANCER.

L. O. BUTLER.

*From the Chester Beatty Research Institute, The Royal Cancer Hospital, London, S.W.3.*

Received for publication April 21, 1951.

THE observation of Brdička (1933) that proteins gave catalytic double waves when added to an ammoniacal cobalt buffer and examined polarographically was soon applied to an examination of the serum proteins, and Brdička (1937) himself was the first to show that differences could be found in this way between normal and cancerous sera. There is no need to relate in detail here the earlier studies on the protein reaction as these have already been adequately reported (Brdička, 1939, 1947 ; Brdička, Novak and Klumpar, 1939), but it will suffice to state briefly that pathological sera after hydrolysis with potassium hydroxide and polarographed in cobaltous buffer show a decreased wave height as compared with normal sera, but after deproteination with sulphosalicylic acid and the filtrates polarographed in cobaltic buffer show an increased wave height. Brdička (1939) found that the latter test, known as the " filtrate test," was more reliable, but the readings from both tests are dependent on the temperature, and on the characteristics of the dropping mercury electrode. Müller and Davis (1947) introduced an index, known as the " Protein Index," derived from the ratio of the two tests multiplied by a constant, which was found to be independent of the temperature and of the capillary characteristics, and also of the age and sex of the subjects.

The fact that the polarographic test is not specific for malignancy has been well established and it is therefore of no use diagnostically, but more recent results indicate that it may be of value as a prognostic tool. Robinson (1948), using the filtrate test, has studied its application in the prognosis of prostate cancers, and has obtained results which are superior to those obtained with the acid phosphatase test normally used for this type of cancer. The purpose of the work to be reported below was to gain further information concerning the usefulness of the method, and to compare it with other methods which might be more applicable in general routine. Over 100 sera have been examined polarographically, and a smaller number examined by means of the " Huggins test " and by the tryptophane-perchloric acid reaction.

Since the publication by Huggins, Miller and Jensen (1949) of the " Huggins test," depending on the relative ease of the thermal clotting of serum in the presence of various concentrations of iodoacetate, many workers have investigated its application (Bodansky and McInnes, 1950 ; Homburger, Pfeiffer, Page, Rizzone and Benotti, 1950). The original authors stated that the " iodoacetate index," obtained by dividing the highest concentration of iodoacetate in which clotting could still occur by the total protein content of the serum, was lower in sera from cases of malignancy when compared with normal sera. The results obtained from an examination of a limited number of sera by means of the modified method (Huggins, Miller and Jensen, 1949, private communication) are reported below.

Increases in the polysaccharide content of sera have been observed by a number of workers in cases of malignancy, tuberculosis, nephrosis, hepatic cirrhosis and pneumonia (Shetlar, Foster, Kelly, Shetlar, Bryan and Everett, 1949). Using the carbazole method Seibert, Pfaff and Seibert (1948) showed that the globulins (particularly the $\alpha_2$-globulin) contained the most of the protein bound polysaccharide. In an attempt to determine the nature of the polysaccharide Seibert and her co-workers (1948) found that the tryptophane-perchloric acid (TPA) test of Cohen (1944) was more useful. Using this reaction the authors claimed a correlation between increases in the polysaccharide and increases in the $\alpha_2$-globulin in cases of tuberculosis and malignancy. Moreover, changes in the TPA readings were found in minimal tuberculosis when no change was observed with the carbazole reaction, and it was suggested that this was probably due to the former reaction being more specific although the latter reaction is probably more sensitive. The identity of the polysaccharide involved in the TPA reaction is not known, but only negligible colour formation is obtained with glucose, mannose, galactose and glucosamine, while a positive reaction is obtained with desoxyribose-nucleic acid and fructose. A few sera have been examined by the TPA reaction and the results obtained are recorded below.

### METHODS.

All cases reported have been from patients at the Royal Cancer Hospital, except for four cases from Ashford Hospital, and for nineteen cases from Mr. D. M. Wallace. The serum was separated from the clot at the earliest moment, and generally stored overnight in the refrigerator. The serum for testing with the Huggins reaction or the TPA reaction may have been stored for longer but not for more than four days.

### *Polarographic.*

The instrument used was a Tinsley pen-recording polarograph. The " filtrate" and " digest " tests and the calculation of the " Protein Index " were carried out according to the method described by Müller and Davis (1947).

(i) *Digest test.*—To 0·5 ml. serum was added 0·5 ml. glass distilled water and 0·25 ml. N potassium hydroxide, the mixture shaken, and allowed to stand for 30 minutes ; 0·05 ml. of the mixture was then added to 10 ml. of the standard cobaltous chloride buffer solution and the mixture polarographed immediately between —0·8V. and —2·0V. The buffer solution was always freshly prepared and made up of 20 ml. 0·008 M cobaltous chloride, 10 ml. N ammonium chloride, 60 ml. water and 10 ml. N ammonia, mixed in that order.

(ii) *Filtrate test.*—To 0·5 ml. serum were added 1·0 ml. glass distilled water, and 0·10 ml. N potassium hydroxide, the mixture shaken, and allowed to stand for 30 minutes. Then 1·0 ml. of 0·8 M sulphosalicylic acid was added, the contents of the tube shaken, and after standing 10 minutes were filtered through a 5·5 cm. No. 5 Whatman filter-paper. To 9·0 ml. of the standard cobaltic buffer solution 1·0 ml. of the filtrate was added and polarographed between —0·8V. and —2·0V.

The standard cobaltic buffer was made up of 10 ml. 0·01 M hexaminocobaltic chloride, 10 ml. N ammonium chloride and 80 ml. N ammonia, mixed in that order.

The digest test was found to give more variable results, and this observation together with the reported instability of the cobaltous buffer led to the replacement of this test by the two following:

(iii) *Whole serum test.*—To 10 ml. of the standard cobaltic buffer 0·1 ml. serum was added and polarographed between —0·8V. and —2·0V.

(iv) *Precipitated serum test.*—To 0·5 ml. serum were added 1·0 ml. water and 1·0 ml. 0·8 M sulphosalicylic acid, the mixture shaken, and after standing 10 minutes filtered through a 5·5 cm. No. 5 Whatman filter-paper. To 9·0 ml. of the standard cobaltic buffer 1·0 ml. of the filtrate was added and polarographed between —0·8V. and —2·0V. A new index was then formulated derived from dividing the value of the filtrate test (Test ii) by the difference between Tests iii and iv and multiplying by ten to bring it to a round number, i.e.,

$$\text{Blood index (B.I.)} = \frac{\text{Test (ii)}}{\text{Test (iii)} - \text{Test (iv)}} \times 10.$$

*Huggins reaction.*

The modified method (Huggins, Miller and Jensen, 1949, private communication) was used. The iodoacetic acid was recrystallized from a mixture of equal volumes of benzene and 80–100° C. petroleum ether. The protein content was determined by the specific gravity method in copper sulphate solutions (Phillips, Van Slyke, Dale, Emerson, Hamilton and Archibald, 1944).

*TPA reaction.*

The method as reported in Seibert, Pfaff and Seibert (1948) was used. Reaction mixtures were made up of 0·25 ml. serum, 0·75 ml. 0·9 per cent w/v saline, 2·0 ml. 0·25 per cent w/v tryptophane and 3·0 ml. 60 per cent w/w perchloric acid. The contents of the tubes were gently mixed, the tubes covered and heated in a boiling water-bath for 10 minutes. Alter cooling quickly they were allowed to stand for 40 minutes and then filtered through a No. 42 Whatman filter-paper. Readings were taken with a Gallenkamp colorimeter using an Ilford 404 filter. Controls were carried out using water instead of serum, and also with 0·25 ml. serum in 5·75 ml. saline, which were unheated. These latter controls usually gave negligible readings.

### RESULTS.

(a) *Polarographic.*

The following tests have been carried out polarographically, 34 tests on serum from 24 normal persons, 129 tests on 87 cancer patients, and 42 tests on 40 patients with non-malignant conditions, and 24 of the cancer patients have been examined at intervals ranging over a period of 1 to 56 weeks.

The results obtained with the malignant cases are shown in Table I, where they are expressed in terms of the numbers of patients giving positive (i.e., pathological) and negative results. The upper limit for normal values was fixed at an arbitrary level below which the majority of the normals were grouped (21 negative, 3 positive). Table I is divided into two groups, and considering the cases placed in Group (a), it can be seen that 93 per cent gave a positive reaction. The only negative readings were in three cases of carcinoma of the

TABLE I.—*Ratio of Positive to Negative Polarographic Results given by Sera from Malignant Cases.*

*Group* (a) : *Excluding Buccal Cavity and Skin Tumours.*

| Site of tumour. | Number of cases. | |
|---|---|---|
| | Positive. | Negative. |
| Alimentary canal | 8 | 0 |
| Breast | 11 | 3 |
| Bronchus | 5 | 0 |
| Ethmoid | 2 | 0 |
| Genital tract (♀) | 5 | 0 |
| Lymphatic | 7 | 0 |
| Prostate | 8 | 1 |
| Kidney | 2 | 0 |
| Femur (fibrosarcoma) | 1 | 0 |
| Cheek (melanoma) | 1 | 0 |
| Bladder | 1 | 0 |

*Group* (b) : *Buccal Cavity and Skin Tumours.*

| Site of tumour. | Number of cases. | |
|---|---|---|
| | Positive. | Negative. |
| Buccal cavity with larynx and pharynx | 8 | 7 |
| Skin | 2 | 2 |

breast and one case of prostatic cancer. The former were (i) a patient with a 5 years' history who had been successfully treated by surgery and by androgen, and who was clinically normal at the time the blood sample was taken ; (ii) a male case who gave a positive reading 2 weeks later ; and (iii) a patient with an advanced tumour who gave a negative reading again 2 weeks later. The negative prostate case had already been receiving stilboestrol treatment.

These cases show a high correlation with the clinical observations, but Group (b) of Table I gives figures for two malignant groups which appear to give an even chance of positive results. Classified in " buccal cavity " are carcinomas of the fauces, palate, tonsil, tongue, mouth, larynx and pharynx, and in " skin " are classified carcinomas of nose and scalp. The reason for the uncertainty in these cases is not clear, but it may be due simply to the fact that the cases of Group (a) had reached the condition at which a positive polarographic reaction was possible by the time a clinical diagnosis could be made, whereas the reverse had occurred with the Group (b) cases, a finding which may be related to the relative ease of diagnosis at an early stage in this latter group. There is no evidence that the method as used at present is sufficiently sensitive to detect disease at its very early stages, although some cases have been received which were originally clinically undiagnosed and yet gave positive polarographic readings. Cancers of the alimentary system are frequently at an advanced stage when diagnosed, and it can be seen from Table I, Group (a) that they have given consistently positive results. However, since negative readings have been obtained with advanced cases of Group (b), it is realized that factors other than the relative ease of diagnosis may influence the relative times at which the positive test is manifested and the symptoms recognized. Taking into account all the results given by malignant cases an 82 per cent correlation with the clinical diagnosis was obtained, although it is perhaps justifiable to consider the Group (b) cases separately.

Of the non-malignant cases 65 per cent gave positive values. The results are given in Table II. Ulcer patients have provided the majority of the cases and these have tended to give high readings. The three negative results related to (i) a patient who had a partial gastrectomy nine years previously and had no clinical evidence of the condition at the time of the blood sample ; (ii) a case who was also suffering from polycythaemia vera ; and (iii) a patient who had a densely adherent ulcer which was invading the pancreas and gave a reading which was near positive.

TABLE II.—*Ratio of Positive to Negative Polarographic Results given by Sera from Non-malignant Cases.*

| Condition. | Number of cases. | |
|---|---|---|
| | Positive. | Negative. |
| Adenoma of thyroid | 0 | 1 |
| Arteriosclerosis | 1 | 1 |
| Coronary disease | 1 | 0 |
| Cystic breasts | 1 | 1 |
| Fibroadenoma of breast | 0 | 1 |
| Hydronephrosis | 1 | 0 |
| Intermittent claudication | 0 | 1 |
| Peptic ulcers | 12 | 3 |
| Prostatic hypertrophy (benign) | 6 | 6 |
| Ulcerative colitis | 1 | 0 |
| Rhinophyma | 0 | 1 |

Some patients in the buccal cavity group also suffered from sepsis, or had given positive Wassermann reactions and yet gave negative readings. Also, some negative cases amongst this group and the skin group had had operations 10 to 12 days previously, so that surgery *per se* did not appear to cause positive results.

In Table III are shown results obtained by the serial examination of patients over a period of time. Except for four readings in the case of the patient Cap. (carcinoma of breast), no disagreement with the clinical observations was obtained. If the patient's condition had not changed, the polarographic readings were unaltered ; if the condition had worsened the readings increased ; if the condition had responded to treatment the readings decreased towards normal. In the case of Cap. great improvement was observed clinically while under androgen treatment (after radiotherapy followed by radical mastectomy), and the polarographic readings altered from positive to normal. However, the last five readings, taken over a period of eight months, gave positive reactions, although it was only at the time of the last reading that a relapse was clinically observed. In this case, therefore, the relapse was forecast by the polarographic readings. The patient Lan. became apparently normal and later relapsed, as reflected in a return to a high polarographic reading. This method of serial examination shows most promise, but demands close co-operation between all concerned.

TABLE III.—*Polarographic Readings Obtained by the Serial Examination of Malignant Cases.*

| Condition. | Name. | Initial. | Filtrate readings. Duration (weeks). 2. | 4–6. | 9–16. | 24–39. | 44–56. | Clinical remarks. |
|---|---|---|---|---|---|---|---|---|
| Cancer of breast | Lan. | 7·75 (+) | 6·5(N) | — | — | 11·9 (+) | — | Very ill. |
| | Ran. | 18·6 (+) | 10·9 (+) | — | — | — | 13·4 (+)$^{47}$ 15·3 (+)$^{56}$ | Deteriorating. |
| | Gos. | 5·75 (N) | 6·0 (N) | | | | | Advanced. |
| | Car.♂ | 4·75 (N) | 7·75 (+) | | | | | Poor outlook. |
| | Cap. | 6·4 (+) | — | 5·25 (N) | 3·75 (N)$^{11}$ 7·5 (+)$^{16}$ | 6·0 (+)$^{24}$ 9·1 (+)$^{38}$ | 5·6 (+)$^{47}$ 8·1 (+)$^{51}$ | Great improvement; no change; relapsed at 51 weeks. |
| | Den. | 17·6 (+) | — | — | 7·8 (+) | | | |
| | New. | 10·75 (+) | — | — | 5·6 (N) | 4·5 (N) | | Improvement. |
| ,, bronchus | Daw. | 16·6 (+) | 13·25 (+) | 8·1 (+) | | | | |
| | Luc. | 10·75 (+) | 10·25 (+) | 9·5 (+) | | | | Some improvement. |
| ,, cervix | How. | 9·9 (+) | — | 10·25 (+) | | | | Died. |
| ,, fauces | Cof. | 10·5 (+) | 9·0 (+) | 9·25 (+) | — | 15·0 (+) | | Very extensive—dying. |
| ,, larynx | Kee. | 3·4 (N) | 5·9 (N) | | | | | Condition good—early. |
| ,, pharynx | Pul. | 9·25 (+) | 5·75 (N) | | | | | Advanced; improved latterly. |
| ,, rectum | Lov. | 17·0 (+) | — | 10·25 (+) | — | 6·8 (+) | | |
| ,, tongue | Arm. | 2·6 (N) | — | — | — | 4·75 (N)$^{24}$ 4·3 (N)$^{36}$ | | Getting worse; more than 5 years' history. |
| | Atk. | 11·5 (+) | — | 12·1 (+) | | | | |
| ,, vocal cord | Kin. | 3·9 (N) | — | 4·1 (N) | | | | |
| Lymphosarcoma | Dor. | 6·75 (+) | — | 7·6 (+) | — | — | 6·3 (+)$^{44}$ 6·3 (+)$^{55}$ | Active initially; improved but liver infiltrated. |
| Melanoma cheek | Del. | 11·5 (+) | — | 18·9 (+) | | | | Died. |
| Unknown | Trap. | 5·1 (N) | — | — | 3·4 (N) | | | Bleeding *per rectum*. Biopsy: inflammatory. |
| | Wor. | 14·25 (+) | — | — | 16·1 (+) | | | Large intraperitoneal mass. |

Indices refer to number of weeks after initial reading.

(b) *Huggins reaction.*

The following tests have been carried out: 4 tests on the serum from 4 normal persons, 23 tests on 20 cancer patients, and 8 tests on 8 patients with non-malignant diseases. Some 75 per cent correlation with the clinical diagnosis was obtained from malignant cases, but again there was no specificity as regards malignancy and non-malignancy. The results obtained are given in Table IV,

TABLE IV.—*Ratio of Positive to Negative Results given by Pathological Sera Examined by the Huggins reaction.*

*Malignant.*

| Site of tumour. | Number of cases. Positive. | Negative. |
|---|---|---|
| Alimentary canal | 2 | 0 |
| Breast | 4 | 2 |
| Bronchus | 1 | 0 |
| Buccal cavity with larynx | 1 | 2 |
| Ethmoid | 1 | 0 |
| Genital tract (♀) | 3 | 1 |
| Lymphatic | 1 | 0 |
| Skin | 2 | 0 |

*Non-malignant.*

| Condition. | Number of cases. Positive. | Negative. |
|---|---|---|
| Adenoma of thyroid | 0 | 1 |
| Cystic breasts | 0 | 1 |
| Hydronephrosis | 1 | 0 |
| Peptic ulcers | 2 | 2 |
| Ulcerative colitis | 1 | 0 |

expressed in terms of the number of patients giving positive and negative readings. Correlating these results with those obtained polarographically there was disagreement in 20 per cent of the cases. For example, the ulcer case previously mentioned who had no clinical evidence of the condition and gave a negative polarographic test gave a positive Huggins reaction. In the other cases there were negatives where the polarograph gave positives. In 40 per cent of the cases no absolute value was obtained as the end-point was outside the recommended iodoacetate concentration range.

(c) *Tryptophane-perchloric acid reaction.*

The following tests have been carried out: 4 tests on the serum from 4 normal persons, 34 tests on 31 cancer patients, and 7 tests on 7 patients with non-malignant diseases. Correlation with the clinical diagnosis of malignancy was almost 100 per cent, and of the non-malignant cases 70 per cent gave positive readings. Disagreement with the polarographic readings was 12 per cent, the TPA reaction giving positives when the polarograph gave negatives. Fig. 1 shows that there is some correlation between the intensity of colour produced and the polarographic readings.

It would seem highly probable that the reactive substance involved in this test is a result of a general tissue protein breakdown, so that the high correlation obtained may not be surprising. Keyser (1949, 1950) investigated patients suffering with burns by this test and obtained results consistent with this view.

DISCUSSION.

From the above results it is obvious that none of the methods used is specific for cancer. Youden (1950) has suggested the use of an index for the comparison of so-called diagnostic methods, which has a value of unity if there are no false positives or false negatives, and a value of zero when the test gives equal proportions of positives for the condition under investigation and the controls. The

FIG. 1.—The relations between the corrected colorimeter readings obtained from the tryptophane-perchloric acid test and the logarithms of the filtrate wave-height and of the blood index.
● = Malignant.
■ = Non-malignant.

application of this index to the results obtained comparing the malignant to the non-malignant patients serves to accentuate the unspecificity of the tests and their uselessness in diagnosis. The polarographic method gives a value for the index of 0·219, the Huggins reaction, 0·250, and the tryptophane-perchloric acid method, 0·253. The value for the Huggins reaction may be compared with that of 0·551 obtained by Homburger, Pfeiffer, Page, Rizzone and Benotti (1950), and with a value of 0·203 calculated from results given by Bodansky and McInnes (1950). However, the index gives no measure of the possible application of the tests in prognosis.

The TPA reaction, although giving high correlation with the clinical diagnosis of cancer, gave readings which bore little relation to the severity of the disease.

Patients showing clinical improvement reflected in the polarographic readings showed no change according to the TPA test. These results, considered together with those of other workers, suggest that any observations on the serum polysaccharide content without any previous fractionation of the serum will only show changes due to the response of the organism to unspecific abnormal conditions. In addition to the pathological conditions mentioned by Shetlar, Foster, Kelly, Shetlar, Bryan and Everett (1949), these authors also showed rises in the serum polysaccharide (determined by reaction with tryptophane (with no perchloric acid) after surgery and after x-ray treatment. It may be concluded therefore that the TPA reaction on whole serum (and perhaps any method measuring whole serum polysaccharide) is unsuitable for use in prognosis.

The Huggins reaction was found to give a lower correlation with the clinical diagnosis. The end-point as described by Huggins was not easily determinable, as it was not always obvious what constituted a " well-defined mass." This difficulty was also noted by Bodansky and McInnes (1950). Also, the end-point often did not occur within the limits of the recommended iodoacetate concentrations—a problem which presumably would have been overcome by using a larger range of concentrations, making the test much more tedious. The results obtained support the conclusions of Bodansky and McInnes (1950) and of Homburger, Pfeiffer, Page, Rizzone and Benotti (1950), which were reached after a much more intensive study of the reaction than has been attempted here, and it can be stated further that the test is too unreliable to be used in prognosis.

The results given in Table III showed that the polarographic readings followed to a very large degree the clinical findings, and it can be stated that within certain limitations this method is useful as a prognostic agent. It can be seen from Table I that apart from the two groups of cancers, a high correlation was obtained and the proportion of false negatives was low. There was, in fact, only one case in the negatives shown in Table I(a), which remains unexplained, since the prostate case was being treated with stilboestrol, and of the breast patients, one was clinically normal and the other case gave a positive reading two weeks later. This last case supports the view that the serial examination of serum gives far greater information than a single test, a view also supported by Brdička (1947) and Robinson (1948). Both positive and negative cases should be followed. A persistently positive test or a negative test becoming positive would indicate that the patient requires investigation, a positive test becoming negative indicates that the patient has improved, while a persistently negative test may be misleading ; this last fact is the main weakness of the method. Many more cases should be followed in order to consolidate these conclusions. The method of serial examination has been used to distinguish between peptic ulcers and carcinomas of the stomach, since the former respond to treatment in a relatively short time with a resulting decrease in the polarographic readings, whereas the carcinoma cases respond slowly if at all (Robinson, 1948).

In the application of the polarographic method to the prognosis of malignancy the limitations of the method should always be borne in mind. It is quite possible that no one test will be satisfactory for all types of cancer, so that a number of tests may have to be used in conjunction. It should also be remembered that serum is subject to very general changes, although the sulphosalicylic acid treatment does provide some fractionation.

Some rigidity of procedure must be adhered to when carrying out the polarographic method. Not only should the hydrolysis with potassium hydroxide and the time of standing after precipitation with sulphosalicylic acid always be for a standard period of time, but the filtrate must be completely cleared with one filtration, since the wave height is increased in the presence of the precipitate and is decreased with successive filtrations.

### SUMMARY.

1. Over 100 sera have been examined polarographically, 32 sera by the "Huggins reaction" and 42 sera by the tryptophane-perchloric acid reaction. None of the methods gave specific results for malignancy.

2. Excluding tumours of the buccal cavity and skin, which gave an equal chance of giving positive or negative readings, polarographic examination gave a 93 per cent correlation with the clinical diagnosis of malignancy. Of non-malignant cases 65 per cent gave positive values.

3. Serial polarographic examinations have been carried out on 19 patients, and good agreement with the clinical diagnosis obtained, except in one case where positive polarographic reactions preceded the clinically observed relapse. It is suggested that the method of serial examinations gives greatest promise in application to prognosis.

4. Examination by the Huggins reaction gave a 75 per cent correlation with the clinical diagnosis for malignancy. The test had no advantage over the polarographic method, and, in fact, was less reliable.

5. A high correlation with the clinical diagnosis of malignancy was obtained with the tryptophane-perchloric acid test, although it is considered too unspecific to be of use in prognosis.

I wish to thank Professor E. Boyland, in whose department this work was carried out. Thanks are also due to Dr. D. A. G. Galton for his criticism and advice and for his assistance in procuring samples of blood from patients of the Royal Cancer Hospital, to Mr. D. M. Wallace and to Mr. F. Goulden for blood samples. The investigation has been supported by grants from the British Empire Cancer Campaign, the Jane Coffin Childs Memorial Fund for Medical Research, the Anna Fuller Fund, and the Division of Research Grants of the U.S. Public Health Service.

### REFERENCES.

Bodansky, O., and McInnes, G. F.—(1950) *Cancer*, **3**, 1.

Brdička, R.—(1933) *Coll. Czech. chem. Commun.*, **5**, 112.—(1937) *Nature* **139**, 330. (1939) *Acta radiol. cancerol. bohem. morav.*, **2**, 7.—(1947) *Research*, **1**, 25.

*Idem*, Novak, F. V., and Klumpar, J.—(1939) *Acta radiol. cancerol. bohem. morav.*, **2**, 27.

Cohen, S.—(1944) *J. Biol. Chem.*, **156**, 691.

Homburger, F., Pfeiffer, P. H., Page, O., Rizzone, G. P., and Benotti, J.—(1950) *Cancer*, **3**, 15.

Huggins, C., Miller, G. M., and Jensen, E. V.—(1949) *Cancer Res.*, **9**, 177.

Keyser, J. W.—(1949) *Nature*, **164**, 889.—(1950) *J. clin. Path.*, **3**, 106.

Muller, O. H., and Davis, J. S.—(1947) *Arch. Biochem.*, **15**, 39.

Phillips, R. A., Van Slyke, D. D., Dale, V. P., Emerson, K., Hamilton, P. B., and Archibald, R. M.—(1944) *Bull. Army Med. Dept.*, **71**, 66.

Robinson, A. M.—(1948) *Brit. J. Cancer*, **2**, 360.

Seibert, F. B., Pfaff, M. L., and Seibert, M. V.—(1948) *Arch. Biochem.*, **18**, 279.

Shetlar, M. R., Foster, J. V., Kelly, K. H., Shetlar, C. L., Bryan, R. S., and Everett, M. R.—(1949) *Cancer Res.*, **9**, 515.

Youden, W. J.—(1950) *Cancer*, **3**, 32.